# A bibliometric analysis of preoperative anxiety research (2001–2021)

**DOI:** 10.3389/fped.2022.938810

**Published:** 2023-01-05

**Authors:** Siyuan Sun, Jingjing Feng, Zhiwei Fu, Zhengyi Lu, Jiafeng Chen, Mingyan Hua, Diansan Su, Juan Gu

**Affiliations:** Department of Anesthesiology, Renji Hospital, Shanghai Jiao Tong University, School of Medicine, Shanghai, China

**Keywords:** preoperative anxiety, bibliometric analysis, hotspot, research front, web of science

## Abstract

Recently, mental health has received increasing attention, particularly preoperative anxiety, which constitutes a bad emotional experience for surgical patients. Many experts have studied preoperative anxiety in terms of its related risk factors, interventions, and postoperative effects; however, there has been no systematic analysis of published articles. This paper presents a bibliometric review of documents related to preoperative anxiety published between 2001 and 2021. A detailed data analysis of 1,596 publications was conducted using CiteSpace and VOSviewer. Since the 20th century, the field of preoperative anxiety has gradually developed; research began around 2000 and has made a huge leap forward since 2016. Developed countries, led by the United States, were the first to conduct research, but preoperative anxiety research in developing countries like Turkey and China has gradually increased and led to an irreplaceable contribution. Intervention has remained the main topic of preoperative anxiety research, and measures have developed from premedication to the provision of education and information. Moreover, the use of advanced equipment such as virtual reality has emerged with great popularity. Based on previous research, the application of virtual reality combined with pediatric patients will become a new research direction.

## Introduction

Preoperational anxiety, or preoperative anxiety, is a common reaction experienced by patients admitted to a hospital for surgery (1). It can be described as an unpleasant state of tension or unease that results from a patient's doubts or fears prior to undergoing a surgery (2).

With medical treatment developments, the estimated total global surgical volume reached levels of up to 312.9 million major operations in 2012 (3). Epidemiological investigations show that the global prevalence of preoperative anxiety among patients undergoing surgery is almost (48%), with the highest prevalence of preoperative anxiety occurring among surgical patients in Africa (56%), followed by those in Asia (54%). Preoperative anxiety level is determined by negative physiological and psychological consequences; examples of these include the fear of an invasive operation, environmental change, being separated from family, worrying about the anesthesia process, and worrying about surgical outcomes such as postoperative pain (4, 5). Among adult patients, preoperative anxiety occurs in 11% to 80% of patients (6). The risk factors for preoperative anxiety include a history of cancer and smoking, psychiatric disorders, negative perceptions of the future, moderate to intense depressive symptoms, high trait anxiety, moderate to intense pain, medium surgery, female gender, and having up to 12 years of education (7). In general, these factors can be categorized as sociodemographic factors, psychosocial factors, and the type of surgery and anesthesia (8). Additionally, having had a previous surgery reduces the risk of preoperative anxiety.

Nonetheless, the influence of preoperative anxiety on patients cannot be ignored. Studies have shown that preoperative anxiety can increase cardiac mortality and adverse reactions during the induction of and recovery from anesthesia (9–11). Moreover, preoperative anxiety also leads to higher levels of postoperative pain, an increased consumption of anesthetics, longer hospital stays, lower quality-of-life, and decreased perioperative satisfaction.

Preoperative anxiety reflects the patient's psychological structure, which can be affected by other people. Too many interventions can be adopted. Pregabalin acting as an antianxiety drug is useful and safe for preoperative and intraoperative anxiety control in patients undergoing surgery (12). Music interventions (13) were provided to reduce antianxiety side effects of drugs. Short-length aromatherapy inhalation is also a worthy recommendation as it acted as an effective intervention for reducing preoperative anxiety in adults (14). Appropriate and effective preoperative education models are promising in terms of decreasing preoperative anxiety in patients scheduled for surgical procedures (15). Therefore, anesthesiologists have the opportunity to reduce the adverse reactions induced by preoperative anxiety by empathetically supporting their patients (16). A patient can be referred to a psychologist for preoperative preparation if a high level of anxiety is detected at an earlier occasion, which is consistent with the guidelines for enhanced recovery after surgeries (17). However, anesthesiologists do not pay great attention to preoperative anxiety and it is not routinely assessed prior to anesthesia administration (18).

Fortunately, in recent years, several research papers about preoperative anxiety have been published. Researchers have investigated medical or nonmedical interventions and the influence and evaluation of preoperative anxiety. It was confirmed that nursing interventions have a positive impact in reducing preoperative anxiety, but the low number of studies and the heterogeneity of the sample size means further research is needed regarding this topic (19). Meanwhile, there remains a lack of quantitative bibliometrics analysis in the preoperative anxiety field. Bibliometric analysis is a popular and rigorous method for exploring and analyzing large volumes of scientific data. It enables the researcher to unpack the evolutionary nuances of a specific field, while shedding light on the emerging areas in that field.

### Research gaps based on literature review

We selected literatures with systematic review and meta-analysis to understand the current status of preoperative anxiety research. However, the studies in these articles mainly focus on: an overview of the effects of the preoperative anxiety, the certain of a intervention to reduce the preoperative anxiety and researches for the specific population such as adults, children or for the specific surgery such as“hip or knee replacement”, “brain surgery”in this filed. These articles can help us understand the degree of research in a certain field of preoperative anxiety, but there is still a lack of an overall grasp of preoperative anxiety research: how preoperative anxiety research have changed in the past, what specific studies have been involved, how these studies were distributed in time and space, what the knowledge framework looks like, what co-cooperation with other studies and what are the hot trends for future research, etc. These can help us to have an overall grasp of the degree of preoperative anxiety research and provide guidance for the future research direction. However, there is lack of such review articles.

### Research questions and research objectives

Considering the research gaps mentioned above, the study addressed the following research question and objectives:

“Do we need to create an overview of status of publications and a clear picture of scientific exchanges?”

The answer to this query is yes. The present study reports a bibliometric review of all papers on preoperative anxiety from 2001 to 2021, based on data obtained from the Web of Science Core Collection with the aid of two tools (CiteSpace and VOSviewer). Given the emphasis on preoperative anxiety, it is necessary to create an overview of status of publications and a clear picture of scientific exchanges in the field because of its significance in helping to master the knowledge before and making research direction decision in the future. Considering the research gaps and intended contribution, the study had the following objectives based on previous literature: publication trends, hotspot shifts, intellectual milestones, and research fronts in the field of preoperative anxiety.

The rest of the paper is organized as follows: Section 2 describes the Materials and Methods. Identifying the articles refined by search strategy. Introducing analytic strategy and the two software programs: Citespace5.8 and VOSviewer1.6.17. Section 3 was the presentation of results base on research objectives: publication trends, hotspot shifts, intellectual milestones, and research fronts in the field of preoperative anxiety. Section 4 was a discussion of the results, followed by the conclusions in Section 5 (20–22).

## Materials and methods

The relevant publications were extracted from the Web of Science (Classic) Core Collection using the following search strategy, and the detailed procedures are drawn in a PRISMA flow chart attached as Supplementary Material:

TOPIC: TS = (*preoperati* near/3 anxiet*) The use of “*” means any group of characters

Refined by: DOCUMENT TYPES:(ARTICLE)

Timespan: 2001–2021.

Language: English

To reduce data bias, we used the Web of Science (Classic) Core Collection, one of the most comprehensive and authoritative databases (23), to perform an advanced search for literature, refined by articles from 2001 to 2021, and downloaded all the required data on January 1, 2022. The downloaded articles were stored in plain text in a newly-created folder titled “Data.” In the meantime, we also created a folder titled “Project” to store the data after analysis. For the bibliometric analysis, we used two software programs: CiteSpace5.8 and VOSviewer1.6.17. CiteSpace is a multi-component, time-sharing, and dynamic citation visualization analysis software that was gradually developed by Professor Chen Chaomei under the background of scientometric, data and information visualization (24); it analyzes literature co-citations and provides co-occurrence analysis between knowledge units, such as author(s), institutions, countries, and regions. VOSviewer is a software tool for creating maps based on network data and visualizing and exploring these maps. Data imported into CiteSpace5.8 were selected by the following options: time-slicing was set to “2001–2021;” the number of years per slice was set to “1;” the selection criterion was set to “g-index;” and the scale factor k was set to “25” for the node type. Only one option at a time was selected for “author,” “institution,” “country,” “reference,” “cited author,” and “keyword.” Data imported into VOSviewer1.6.17 were used to create the term maps by using the following options: “Create a map based on bibliographic data,” “read data from bibliographic database files,” “type of analysis: co-occurrence,” “unit of analysis: all keywords,” “counting method: full counting,” and “minimum number of occurrences of a keyword: 25.” (23)

We began our analysis with a comprehensive overview of preoperative anxiety publications. First, we drew a chart illustrating the number of annual publications. Using VOSviewer, we noted the distribution of research on preoperative anxiety across national and regional research institutions over time. Meanwhile, we used CiteSpace to visualize author co-citation relationships and the cooperation across disciplines in preoperative anxiety.

CiteSpace used to identify the bursts of keywords is a valuable indicator of most active research topics (25). Using VOSviewer cluster analysis, we clustered preoperative anxiety keywords based on their citation frequencies. According to the cluster results, we acquired the hotspots in this field. The software colors keywords differently based on the year of publication. Thus, we were able to visualize changes in hotspots over time, as well the research's center of gravity.

Intellectual milestones are the most influential research studies in a particular field. By reading these articles, we acquired a deeper understanding of the progress of research into preoperative anxiety. In the CiteSpace co-citation cluster analysis, references with betweenness centrality >0.1 had purple circles. we focused on articles with high betweenness centrality, or nodes with thick purple circles; these articles are the bridges that form correlations between articles. Intellectual milestones are regarded as an important theoretical basis to guide the formation of new research results.

A research front is the foundation of scientific research; trends in research fronts reflect the direction of scientific research. Through burst detection of the cited literature in the co-citation network, articles can be ranked in terms of the strength of their citation bursts, illustrating citation frequency increases over a short time. References with the strongest citation bursts meant that the research had recently received a high level of attention and had a large influence on the field. In the meantime, these influential articles may also form the basis of future research. The co-citation network is based on the analysis of the co-citations of downloaded references; each citation is replaced by a small node and the network is formed by connecting lines between related citations, wherein nodes with red circles represent bursts. Generally, the thicker the red circles, the stronger the bursts and the more likely the citation is to become a research front. Nonetheless, using only the co-citation network to detect the scientific research front has some limitations. For example, ongoing studies are not counted and these studies may reflect the research fronts to some extent. ClinicalTrials.gov was selected to identify ongoing studies on preoperative anxiety by searching studies with recruiting, not yet recruiting, or active, but not recruiting.

## Results

### An overview of preoperative anxiety publications

**Figure 1:**
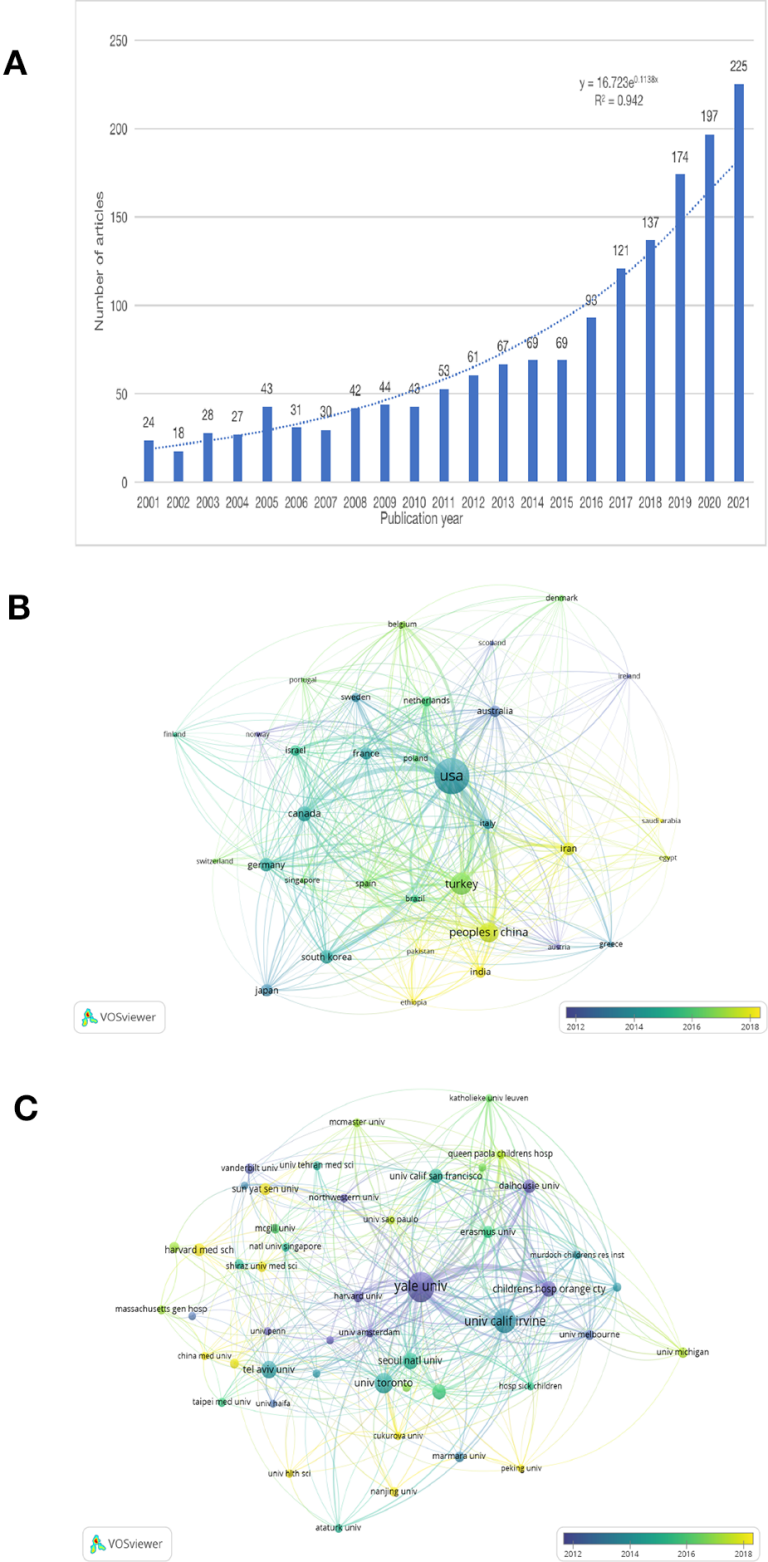
An overview of trends in preoperative anxiety publications. (**A**) Quantitative growth process of studies concerning preoperative anxiety over a period of 21 years. (**B**) Network of countries, created by VOSviewer. Node size illustrates the occurrence times of the countries. (**C**) Network of institutions, created by VOSviewer. Node size illustrates the occurrence times of the institutions.

**Figure 2:**
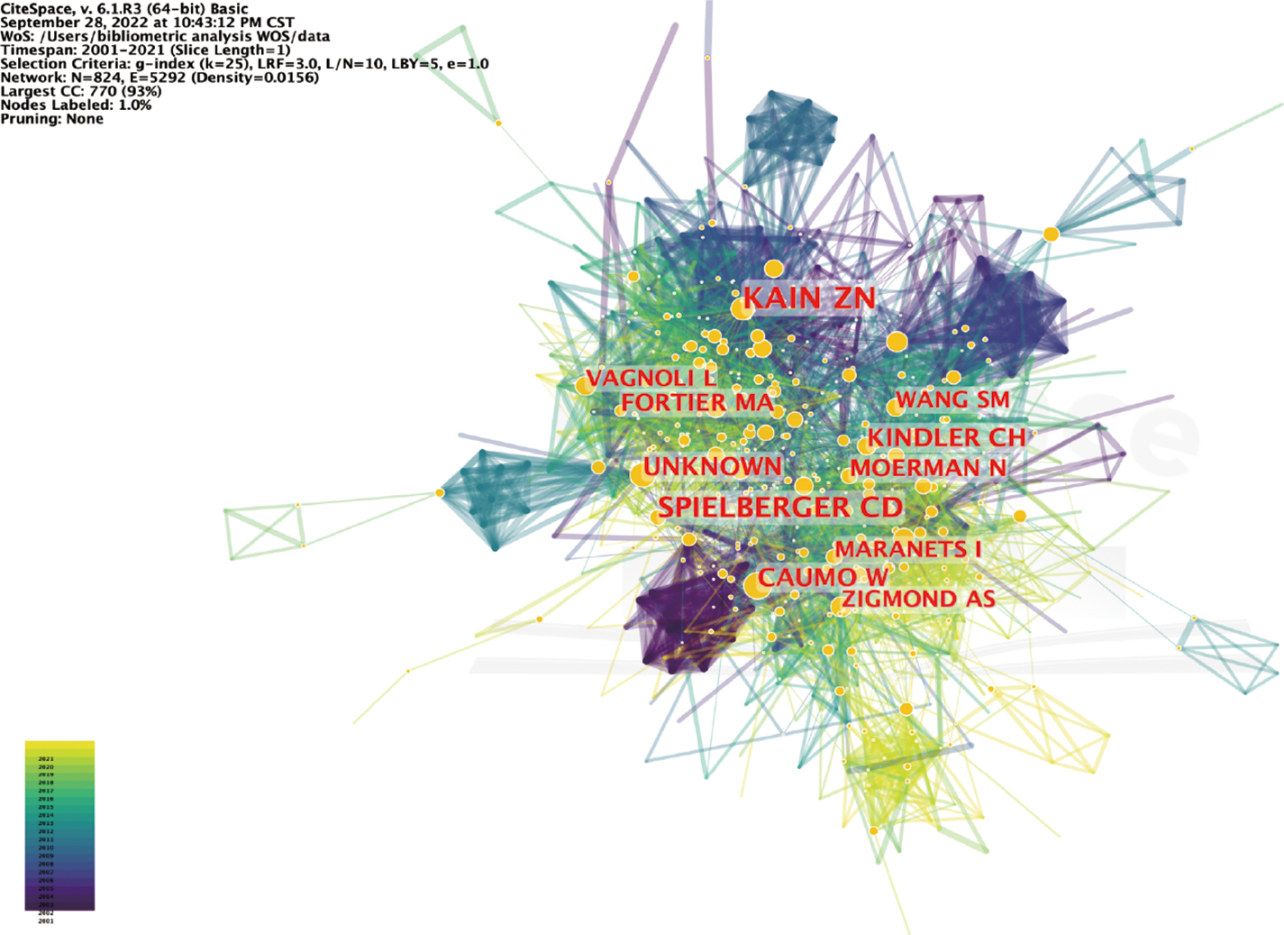
Author co-citation analysis of preoperative anxiety.

**Figure 3:**
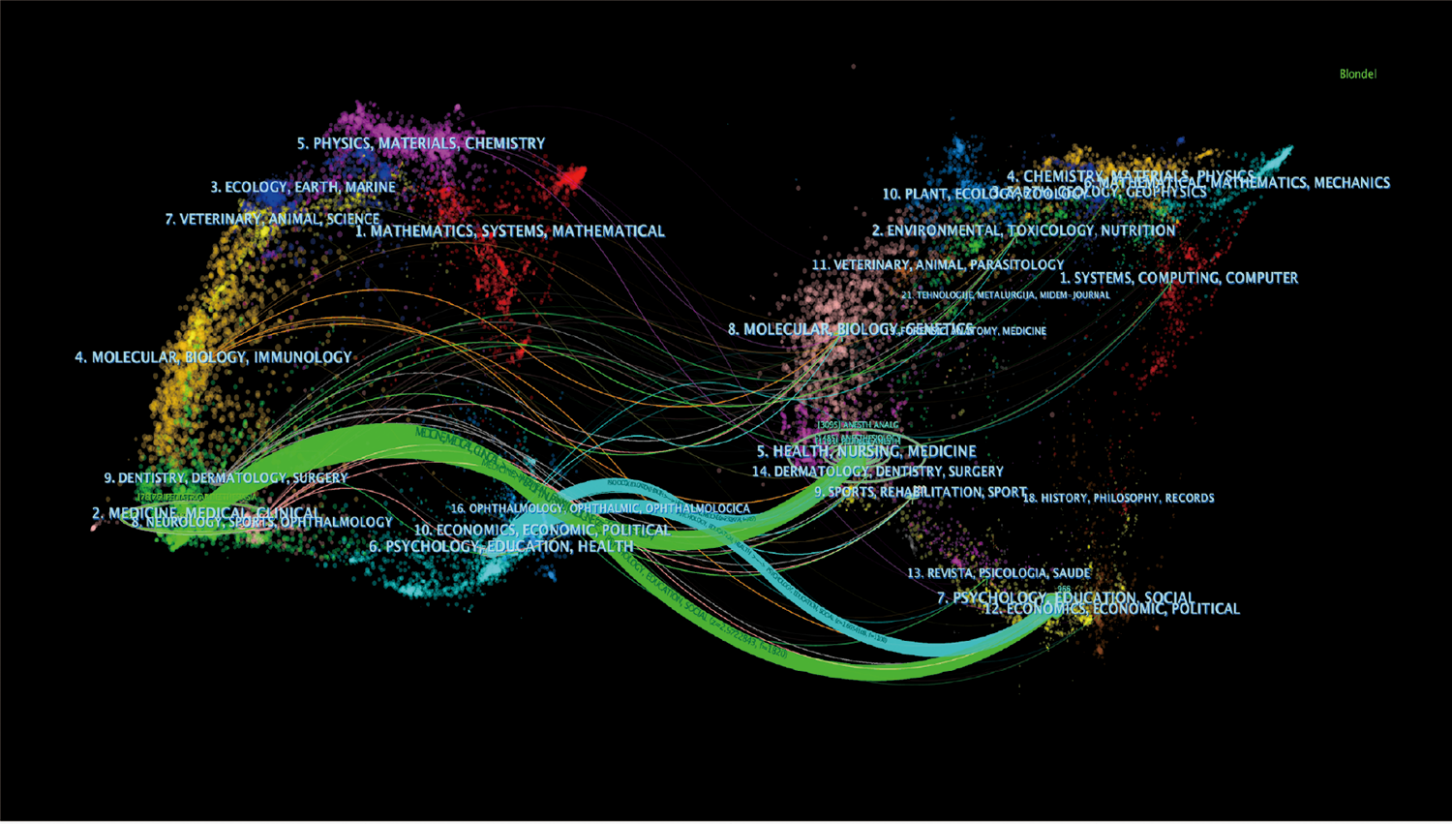
A dual-map overlay of the literature about preoperative anxiety.

We conducted a preliminary analysis of preoperative anxiety publications from a macroscopic perspective. The Web of Science (Classic) Core Collection generated 1,596 publications, as shown in Figure 1A, illustrating that, in the past 21 years, the number of publications related to preoperative anxiety increased unevenly from 2001 to 2021. Prior to 2016, the growth rate was very slow, increasing by an average rate of 3.4 articles each year; however, there was a sharp increase from 2015 to 2016, with an increase of 24 articles compared to the same period over the previous year. After 2016, the growth rate reached a high level, peaking at 225 articles in 2021. The equation presented in Figure 1A can predict the documents published yearly having an R-square of 94% (26). We used VOSviewer software to visualize the networks of citation information for countries (Figure 1B) and institutions (Figure 1C). The highest number of preoperative anxiety studies was conducted in the United States, which accounted for both the early studies and the largest proportion of studies. Canada, Germany, and other developed countries began studying preoperative anxiety before developing countries like Turkey, China, and India; however, developing countries are becoming increasingly influential with respect to preoperative anxiety research, and are expected to become essential areas for future studies. With respect to the institutional network (Figure 1C), Yale University appeared to play a significant role in this area of research, followed recently by the University of California, Irvine. Moreover, Nanjing University and China Medicine University have become new institutions conducting preoperative anxiety research, which is consistent with the results illustrated in Figure 1B. We used CiteSpace software to see the author co-citation (Figure 2) and the cross-disciplinary collaborations (Figure 3) in the preoperative anxiety field. As shown in Figure 2, color correspondence is based on the author's appearance, and the color depth represents time. The nodes represented the authors' co-citation strength and gathered in the 2000s. Our results indicate Caumo W, Unknown, and Kain ZN had the three largest nodes, suggesting that they had a great influence on other authors and could be regarded as the founders of this study in the field of preoperative anxiety. In Figure 3, there were two large clusters distributed in the two sides of this picture. The nodes in Figure 3 represented different references, which were summarized by CiteSpace into different subjects. The cluster on the left represents the references that had been downloaded and the cluster on the right represents references cited by the references that were left. The curves between them represent their connection. The thicker the curve, the stronger the connection. In the cited references, there were two main subjects (5. HEALTH, NURSING, MEDICINE and 12. PSYCHOLOGY, EDUCATION, SOCIAL) that had the strongest connection with downloaded references, which was subject (2. MEDICINE, MEDICAL, CLINICAL). This means there was some cooperation in these fields. We also found that there were some curves between the other subjects. Although their curves were too slender to ignore their connection, they confirmed that numerous different cited reference subjects were the basis of all the downloaded references, which was subject 2. (MEDICINE, MEDICAL, CLINICAL).

### Hotspots in preoperative anxiety research

**Figure 4:**
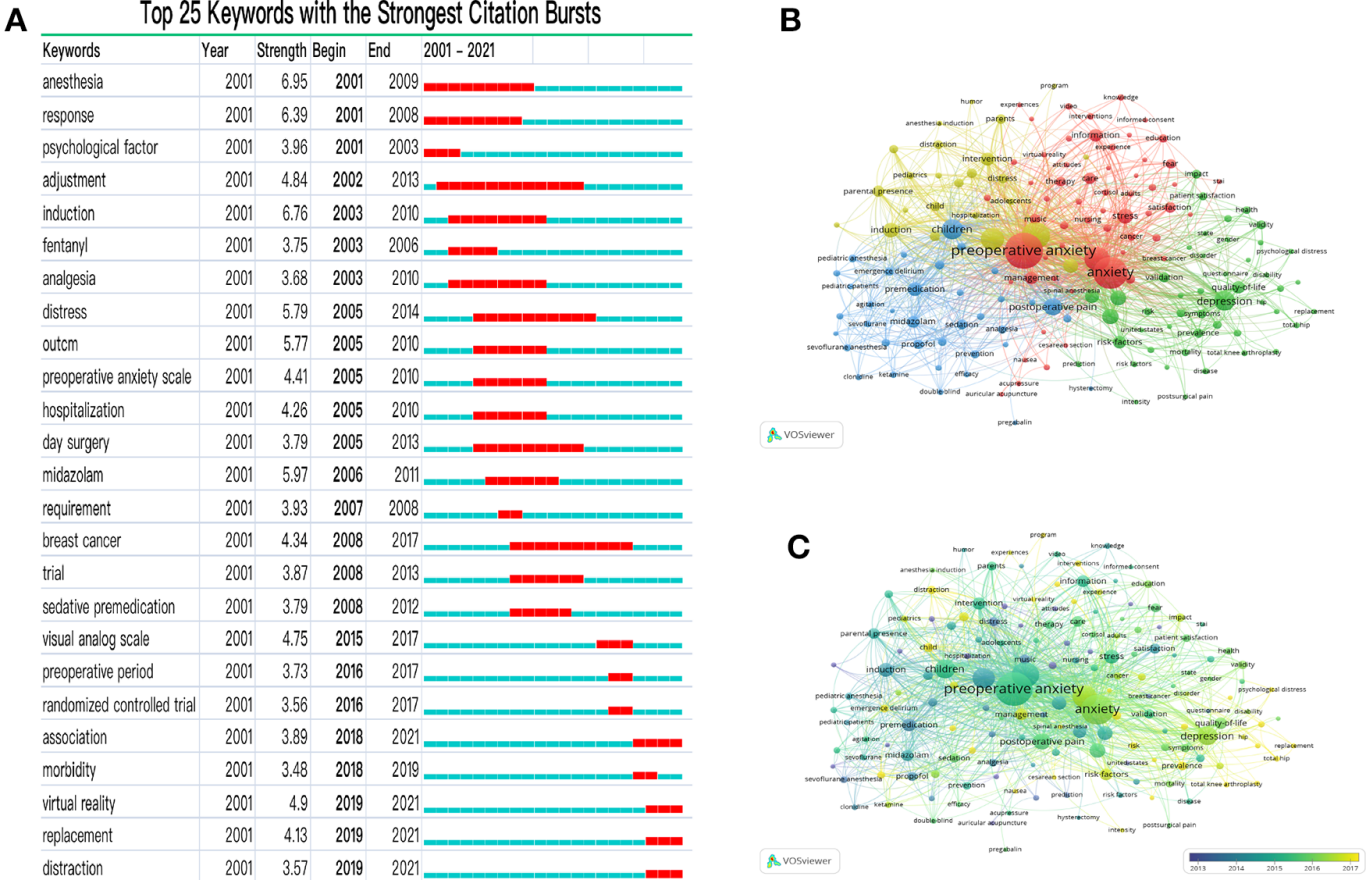
Preoperative anxiety hotspots. (**A**) Top 25 keywords with the strongest citation bursts. (**B,C**) Term co-occurrence network, created using VOSviewer: B) network visualization, and C) overlay visualization. Circle size reflects the occurrence times of the terms. In B, circles are colored according to their clusters. In C, circles are colored according to their average occurrence time.

Hotspots represent the topics that most frequently appear in relevant studies; in other words, hotspots are areas that receive the most attention from scholars, which highlights the focus of preoperative anxiety research. In CiteSpace, burst detection revealed the keywords whose frequencies increased abruptly over time, such that the burst of keywords is a valuable indicator of most active research topics (25). Figure 4A was created by CiteSpace software selecting the top 25 keywords with the strongest citation bursts. In Figure 4A the time interval is depicted by a blue line, whereas the red line represents the period of time in which a keyword was detected as having a burst, over the span from the beginning to ending years. Prior to 2015, the keywords with the strongest citation bursts were “anesthesia,” “response,” and “adjustment.” After 2015, the keywords with the strongest citation bursts were “virtual reality,” “replacement,” and “distraction.”

We used VOSviewer to construct the term co-occurrence network and acquire more information between different keywords. Figure 4B and Figure 4C were created by VOSviewer selecting “Network Visualization” and “Overlay Visualization.” In Figure 4B, each circle represents a keyword, and related keywords are grouped into one item with one color. items are roughly divided into four clusters, and the internal relationships between these four clusters are apparent; these clustered are centered around: 1) using medicine during the perioperative period (blue); 2) preoperative anxiety in pediatric patients and parents (yellow); 3) educational and informational interventions (red); and 4) recovery and outcomes (green). Based on Figure 4B, the color of the circle in Figure 4C was changed according to their average occurrence time, thus enabling the identification of different hotspots from different time periods. Based on the Figure 4C, the application of medicine mostly occurred in the past, whereas non-premedication and informational interventions are new hotspots. These findings are consistent with the burst detection results.

### Intellectual milestones in preoperative anxiety research

**Figure 5:**
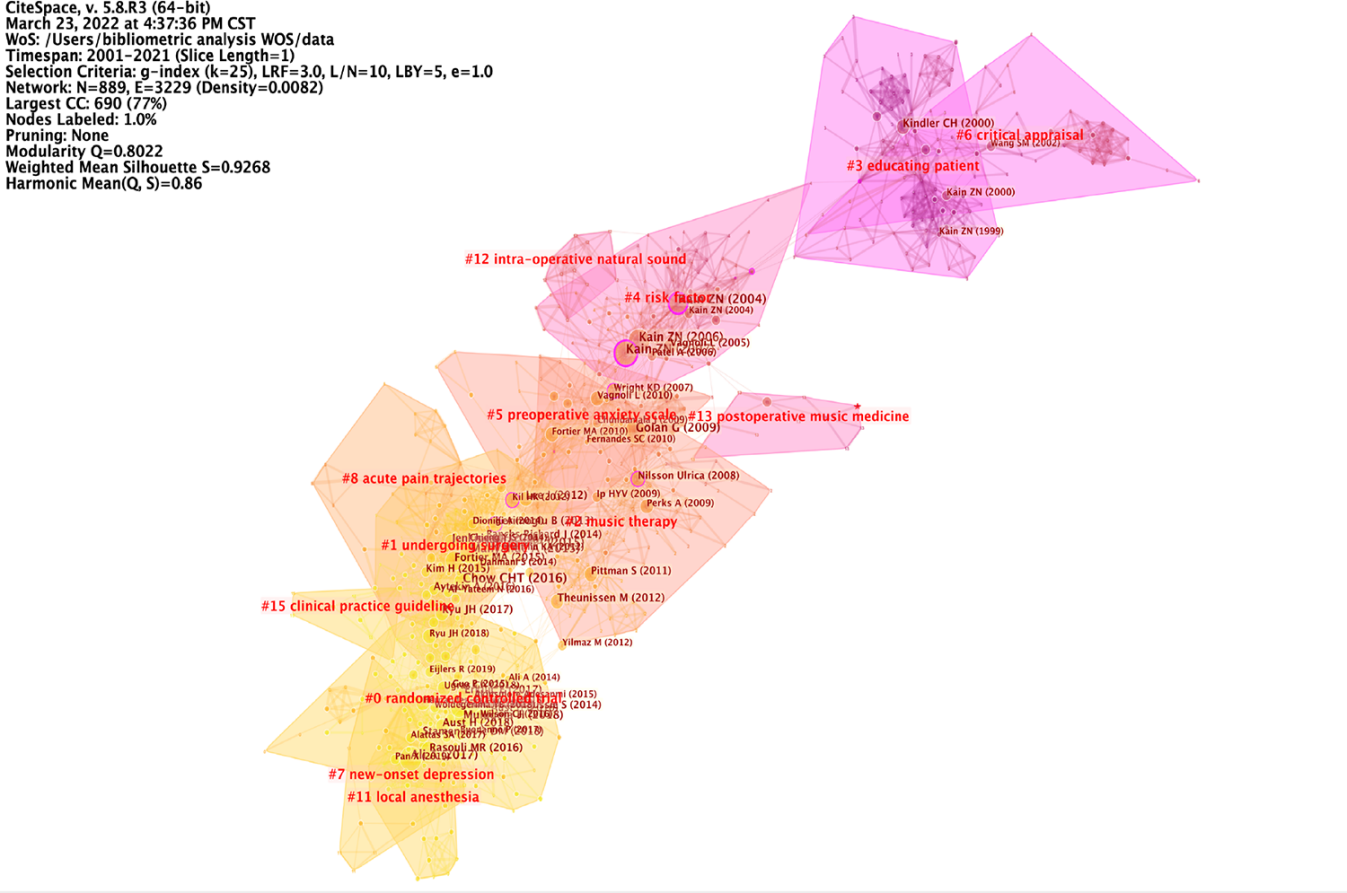
Referrence co-citation analysis of preoperative anxiety. A co-citation network landscape view was generated by a g-index (k = 25) between 2001 and 2021. The network has a modularity of 0.80, suggesting that the specialties were clearly defined. The weighted mean silhouette score was 0.92, suggesting that the homogeneity of the network was high. The nodes in the network were cited articles and the links refer to the co-citation relationship between the cited articles. Node sizes are proportional to the articles’ cited times. The areas of different colors indicate the time when co-citation links in those areas appeared for the first time. Nodes with purple circle represent betweenness centrality > 0.1.

Intellectual milestones are significant research studies. They are the result of quantitative to qualitative change based on previous research. Meanwhile, intellectual milestones in one field can have great impacts with respect to opening another field of research. these articles are the bridges that form the correlation between two unconnected clusters, indicating the “turning point” in CiteSpace, called “intellectual milestones” (Figure 5). In the CiteSpace reference co-citation analysis, there is an index called “betweenness centrality,” which refers to the connection strength between two unrelated things through this medium. Every node represents a cited reference in this analysis. References with >0.1 betweenness centrality means that they have a close link between two unrelated articles. These references have a great potential to be cited as a knowledge base. They are represented by purple circles around the node in Figure 5 and can be called intellectual milestones.

As shown in Table 1, intellectual milestones almost gathered in the 2000 s. We selected the first five articles with the strongest betweenness centrality measures for further analysis (Table 1); these articles all investigated preoperative anxiety in pediatric patients and their parents, a bridge between preoperative anxiety and pediatrics. Among these articles, the first two articles were written by Kain ZN, and have the strongest betweenness centralities of 0.26 and 0.24.

**Table 1:** Five articles with the strongest betweenness centrality.

Article number	Article title	Betweenness centrality	Year
1	Preoperative Anxiety and Emergence Delirium and Postoperative Maladaptive Behaviors (27)	0.26	2004
2	Family-centered Preparation for Surgery Improves Perioperative Outcomes in Children (28)	0.24	2007
3	Non-pharmacological interventions for assisting the induction of anaesthesia in children (29)	0.16	2009
4	The introduction of a pediatric anaesthesia information leaflet: an audit of its impact on parental anxiety and satisfaction (30)	0.16	2002
5	Anesthesia Induction Using Video Glasses as a Distraction Tool for the Management of Preoperative Anxiety in Children (31)	0.14	2013

The first article (27), Kain ZN investigated the negative effects of preoperative anxiety on pediatric patients by increasing the degree of anxiety then evaluating any bad effects, such as emergence delirium and postoperative maladaptive behaviors. The second article (28) integrated literature in both the anesthesia and psychological milieus, in which the authors developed a behaviorally-oriented perioperative preparation program for children undergoing surgery that targeted the family, illustrating that obtaining family-centered preparation is effective in reducing preoperative anxiety and improving postoperative outcomes.

The remaining three articles had betweenness centralities below 0.2, and their main content related to relieving the preoperative anxiety of children or parents *via* non-drug interventions. The paper by Yip (29) assessed the effects of non-pharmacological interventions in assisting the induction of anesthesia in children by reducing their anxiety and distress or increasing their cooperation. The article by Bellew (30) described a randomized controlled trial to detect whether pediatric anesthesia information leaflets have an impact on parental anxiety and satisfaction; this research illustrates that such a practice works, but should not replace verbal communication with medical staff, who remain important sources of information. Finally, the article by Kerimoglu (31) compared the use of video glasses and midazolam with respect to managing preoperative anxiety in children.

### Research fronts in preoperative anxiety

**Figure 6:**
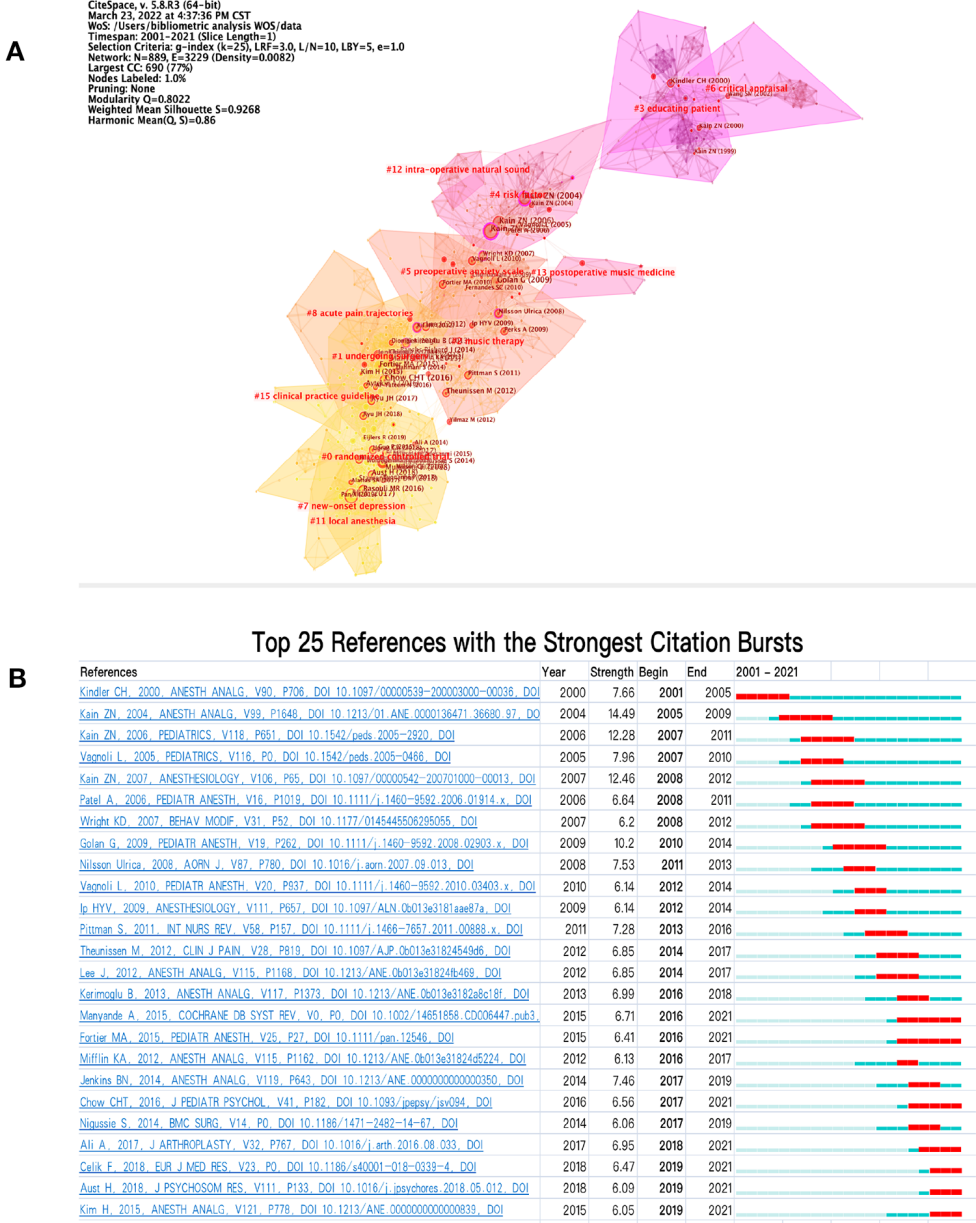
Research fronts in preoperative anxiety. (**A**) A co-citation network landscape view was generated by a g-index (k = 25) between 2001 and 2021. The network has a modularity of 0.80, suggesting that the specialties were clearly defined. The average silhouette score was 0.92, suggesting that the homogeneity of the network was high. The nodes in the network were cited articles and the links mean the co-citation relationship between cited articles. Node sizes were proportional to the times the articles were cited. The different color areas indicate the time when co-citation links in those areas appeared for the first time. Nodes with red circles represent strong citation burst; the thicker the circle, the more frequently the article was cited in a short time. (**B**) Top 25 references with the strongest citation bursts.

Research fronts represent the newest direction of the research. We used CiteSpace software to detect the burstness of references (Figure 6A) and selected the top 25 references with strongest citation bursts (Figure 6B). By a sudden increase in citation frequency over a short period of time (Figure 6A), we can get the focus of attention of experts and scholars. As shown in Figure 6B, we selected the top 25 references with the strongest citation bursts; before 2010, there were 10 articles published, and only 4 articles had the strongest bursts over 10. Although the strength of bursts articles published after 2010 were smaller than those published before 2010, the total number of articles increased by 15; most of these articles describe studies of preoperative anxiety in children, with interventions including clown doctors (32, 33), sedative premedication, parental presence during anesthetic induction, behavioral preparation programs, music therapy, and acupuncture (34). Although articles with the strongest citation bursts were published before 2010, more than half of the top 25 references were published after 2010.

In addition, we use ClinicalTrials.gov to identify ongoing studies on preoperative anxiety. As of the search date of January 1, 2022, 35 studies were recruiting, not yet recruiting, or active, but not recruiting. The descriptions of these 35 studies on preoperative anxiety provided conclusions that were approximately consistent with the CiteSpace results. As shown in Table 2, these articles were divided into three main themes: 1) intervention; 2) the effects of preoperative anxiety; and 3) the measurement and evaluation of preoperative anxiety. Firstly, 25 out of 35 studies were intervention articles with respect to preoperative premedication, preoperative education & information & knowledge, music, multimedia, the application of virtual reality, and other traditional Chinese medicine therapies, such as lavender aromatherapy and auriculotherapy. Among the intervention studies, 9 out of 25 concerned the application of virtual reality technology, especially the application of virtual reality technology in pediatric surgery to reduce the incidence of preoperative agitation and anxiety, which may become a future hotspot in this field. Moreover, six studies investigated the influence of preoperative anxiety, mainly involving early postoperative cognitive dysfunction, postoperative analgesic application, postoperative pain, and sleep quality. The remaining three studies concerned preoperative anxiety level measurements, including behavioral assessments, and physiological changes in the heart and pupils. Additionally, one study has investigated the status of preoperative anxiety during COVID-19, which occurred in 2019; this is clearly in line with the current research background.

**Table 2:** Clinical trials categorized according to their research theme.

Theme number	Research theme	Count
1	Intervention of virtual reality application	9
	Intervention of education, information, and knowledge	5
	Intervention of premedication	3
	Intervention of traditional Chinese medicine therapy	2
	Other patient interventions	6
2	The effects of preoperative anxiety	6
3	The measurement and evaluation of preoperative anxiety	4

## Discussion

With economic and societal development, health is not only limited to physiology; psychological problems, including preoperative anxiety, are receiving increasing levels of attention. Research on preoperative anxiety has rapidly increased. This article aimed to provide a detailed bibliometric analysis of global preoperative anxiety research published between 2001 and 2021. This analysis reflects the status of research in the field and builds a foundation for future research. The disciplines related to this filed were medicine, health, nursing, education, and the different interventions to reduce preoperative anxiety were confirmed.

The number of publications on anxiety increased year over year from 2001 to 2021, especially after 2016 (Figure 1A); this may be related to increasing psychology research in developing countries such as Turkey, China, and India, along with their economic development. Nanjing University and China Medicine University became new institutions that provided a great contribution to preoperative anxiety research in China. In the past two decades, the United States has led the world in terms of the number of research studies and publications on preoperative anxiety (Figure 1B), with Yale University contributing the largest proportion of research in this field (Figure 1C). Canada, Germany, and other developed countries began preoperative anxiety research at earlier dates than developing countries, confirming that the development of psychological development is inseparable from economic development. Kain ZN had a great influence in the field of preoperative anxiety, which was consistent with the intellectual milestones published by him. The study of preoperative anxiety mainly focused on the fields of medicine, education, nursing, and psychology.

Research hotspots and intellectual milestones allow for a rough understanding of study priorities during different time periods as well as the theoretical foundations that have influenced this field. When the field first started, researchers focused on the application of medicine in perioperative patients. For instance, melatonin, given as a premedication, can reduce preoperative anxiety in adults. Additionally, intraoperative propofol sedation can alleviate patients' anxiety, improve comfort level, and lower physiological stress during surgeries under epidural anesthesia. These examples demonstrate that, in the past, the initial thoughts with respect to alleviating the adverse effects of preoperative anxiety on patients were to use drugs and other medical methods as interventions; this explains the keywords “response” and “adjustment” having the strongest citation bursts in the 2000 s. However, with the increase in the types of surgery and the expansion of the age range of patients, research on new interventions and groups is growing. Meanwhile, patients with preoperative anxiety may benefit from multimodal analgesia, including non-pharmacological methods, such as cognitive therapy, music therapy, and relaxation (17). This intervention shift has greatly facilitated the development of preoperative anxiety research. During the 2010 s, education, information, satisfaction, and quality-of-life became new hotspots; these research studies have focused on non-medical interventions and patients’ perioperative feelings.

Intellectual milestones were mainly concentrated in the 2000 s; during the 2010 s, there was only one article with the lowest centrality out of the top five articles with the highest centrality. All these articles revolved around the theme of children, perhaps due to them being a special population, their psychological structure, and the reflection of their emotions differing from those of adults. Parent anxiety differs from the preoperative anxiety experienced by the pediatric patients themselves; therefore, in the study of preoperative anxiety, “children,” “pediatric,” “parents” anxiety” have been and will continue to be research hotspots. In addition, with respect to the cluster of early research organization, there were at least four children's hospitals or children's research institutes. Their clinical resources promoted the development of preoperative anxiety studies in children. We selected five articles with high centrality, and the two most influential articles were written by Kain ZN, an anesthesiologist and pediatrician whose research focuses on stress, pediatric pain, neuropsychology, and value-based care, is known internationally for his pioneering work transforming surgical care. He has significantly improved perioperative clinical services, and over the past 20 years, his research has also focused on surgical care preparation and child home care. In around 2000, Kain ZN published a number of articles related to preoperative anxiety and two of his articles (27, 28) have had the highest impact on the field; both articles investigated preoperative anxiety in pediatrics. As a bridge linking psychology and pediatrics, these two articles became a milestone in the study of preoperative anxiety, opened a new research direction for the field, and laid a theoretical foundation for subsequent research. Meanwhile University of California became one of the most significant institutions attributed to Kain ZN as the head of the Department of Anesthesiology at the University of California.

The visualization of the research front can aid in determining the main research direction and most important papers in the research of unknown fields. The research front refers to an article that received an increase in citation frequency over a short period of time. As such, it has the large potential to be a theoretical basis of a future intellectual milestone. In our analysis, we found that articles with strong bursts were concentrated prior to 2010, mainly because the intellectual milestones were mainly distributed in the 2000s and were frequently cited. Although the bursts were lower than prior to 2010, the number of articles published since 2010 was higher than prior to 2010, indicating that research on preoperative anxiety has recently become more active with increasingly significant influence. Most of these articles are studies of pediatric preoperative anxiety, and two articles (35, 36) are concerned with measuring adult preoperative anxiety using assessment tables. As such, the focus of this research is still on the preoperative anxiety in pediatric.

Children, especially infants, are unable to accurately describe their current feelings. They usually choose to cry and make uncooperative movements to express their physical and psychological discomfort due to separation from their parents as well as entering the unfamiliar surroundings, medical staffs have difficult in taking operation and make decision on symptomatic treatment. On the other hand, children are more vulnerable than adults. Because of their special physiological structure, they have weaker ability to reserve oxygen. The incidence of intraoperative hypoxemia is high and related to age. It was reported that there were over10% incidences happened to 8-to year-old children and over 50% in neonates. Respiratory severe critical events in pediatric anesthesia occur with an incidence of 3.1% (37). In terms of the anesthesia process, it is more likely to appear hypoxia and other bad conditions, which increases the risk factor in surgery, and then increase the anxiety before surgery. As to children under the age of 18, parents are the main caregivers, children have a strong dependence on their parents. Because of parents being used to be depended on, when children accepted the surgery on their own, parents and children will also develop separation anxiety. The existence of this separation anxiety coupled with the worries of the surgery and anesthesia increase the preoperative anxiety. Besides the field of pediatric surgery developed as a result of the need for specialized treatment of uncommon and complex congenital anomalies and childhood surgical diseases. Its professionalism and necessity prompt the pediatric surgery gradually developed into a distinct surgical specialty (38). All above account for why the research on preoperative anxiety in pediatric has been a hotspot in the past and even in the future.

Using ClinicalTrials.gov, we detected ongoing studies on preoperative anxiety, and noted that preoperative anxiety interventions remain the focus of research; this is expected since preoperative anxiety has a variety of perioperative side effects that cannot be ignored. With the development of society, interventions have shifted from medicine to information and technology. Recently, increasing attention has been paid to virtual reality (VR), which can be applied to different patients and surgeries, especially in pediatric patients.

Today is an information society, the development of computer technology will push the artificial intelligence applied to all fields, medicine is no exception. Virtual reality (VR) is a computer technology that provides the feeling of being immersed in a simulated three-dimensional (3D) world where the user may interact with the virtual environment (39). VR technology has an effect on reducing acute procedural pain and providing anxiety relief pain (40). The equipment used is inexpensive, non-invasive, easily available, and transportable, and may be used by anyone (41). This process can help patients shift from passive acceptance to active participation treatment, increasing the awareness of their psychological changes. Meanwhile, the application of VR technology requires no extra labor at the time of ensuring the effect of reducing preoperative anxiety. It not only alleviates the shortage of medical resources caused by the shortage of clinical nurse, but also promotes the efficient operation of clinical work. The combination of VR technology and pediatrics may become a landmark study on preoperative anxiety in the future.

Our research still has some limitations. First, our data were extracted from Web of Science, which means that relevant research from other databases may have been missed. Additionally, the methods of almost all bibliometric analyses are based on the refinement of articles, ignoring other types of documents. For example, by analyzing the research results of reviews, we can also have an overall understanding of the research on preoperative anxiety, the research status and shortcomings of preoperative anxiety, and the future research directions. Moreover, our search strategy was in English, excluding any non-English research; this is particularly important since many developing countries (e.g., China, Turkey) have achieved great progress in preoperative anxiety research, and their exclusion may introduce bias.

## Conclusion

We conducted a bibliometric review of preoperative anxiety articles and analyzed publication trends, hotspots, intellectual milestones, and research fronts. We get the overview of preoperative anxiety publication. There were 1,596 publications during 2001–2021. There was a sharp increase from 2015 to 2016, peaking at 225 articles in 2021. United States had the highest number of preoperative anxiety studies followed by some developed countries: Canada, Germany, etc. Yale University appeared to play a significant role as an institution followed by the University of California, Irvine. Caumo W, Unknown, and Kain ZN could be regarded as the most influential authors in this filed. Kain ZN had the huge influence on this field with his famous studies focus on preoperative anxiety in pediatric, which was regarded as intellectual milestones. The keywords had changed over time, the application of medicine mostly occurred in the past, whereas non-premedication and informational interventions are new hotspots. Research on preoperative anxiety in pediatric would remain a hotspot for a long time in the future. Besides because of the development of the artificial intelligence, the application of VR technology combined with pediatric may lead the field in a new future direction.

## Data Availability

The original contributions presented in the study are included in the article/**Supplementary Material**, further inquiries can be directed to the corresponding author/s.
