# Supplementary figures and images for: A bibliometric analysis of preoperative anxiety research (2001–2021)
Source: Front Pediatr. 2023 Jan 5;10:938810. doi: 10.3389/fped.2022.938810 (PMC9850090; doi:10.3389/fped.2022.938810)

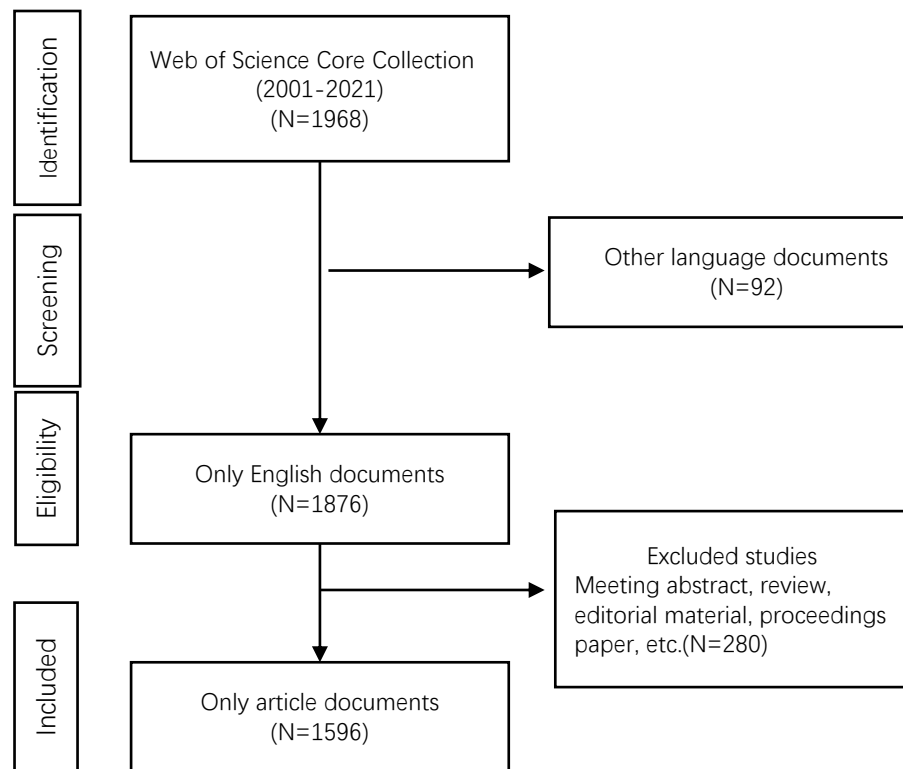

sFigure1:Flow chart

Supplement: Supplementary file 1 [file Datasheet1.pdf]
